# Whole-Genome Sequence of *Salmonella enterica* subsp. *enterica* Serovar Typhimurium Strain UPM 260, Isolated from a Broiler Chicken in Perak, Malaysia

**DOI:** 10.1128/genomeA.01272-17

**Published:** 2017-11-16

**Authors:** Najwa Syahirah Roslan, Shagufta Jabeen, Nurulfiza Mat Isa, Abdul Rahman Omar, Mohd Hair Bejo, Aini Ideris

**Affiliations:** a Institute of Bioscience, Universiti Putra Malaysia, Serdang, Selangor, Malaysia; b Faculty of Veterinary Medicine, Universiti Putra Malaysia, Serdang, Selangor, Malaysia; c Faculty of Biotechnology and Biomolecular Sciences, Universiti Putra Malaysia, Serdang, Selangor, Malaysia

## Abstract

*Salmonella enterica* subsp. *enterica* serovar Typhimurium is one of several well-categorized *Salmonella* serotypes recognized globally. Here, we report the whole-genome sequence of *S*. Typhimurium strain UPM 260, isolated from a broiler chicken.

## GENOME ANNOUNCEMENT

The majority of *Salmonella enterica* infections recorded in humans are foodborne, with *S. enterica* serotypes Enteritidis and Typhimurium being the two most reported serovars (1). The incidence of *S. enterica* serovars differs considerably according to locality, district, region, and country. Hence, detailed surveillance and identification of the prevalent *S. enterica* serovars in humans and poultry should be implemented to establish a control program for an area. True to its name, *S. enterica* is associated with intestinal diseases of chickens, turkeys, and ducks worldwide (2). Multiple signs can be observed in the early onset of infection, including dejection, ruffled feathers, closed eyes, diarrhea, vent pasting, and loss of appetite and thirst. Postmortem lesions include enteritis, focal necrotic intestinal lesions, foci in the liver, unabsorbed yolk, cheesy cores in the caeca, and pericarditis (3).

The *S. enterica* subsp. *enterica* serovar Typhimurium strain UPM 260 was isolated from a broiler chicken in Perak, Malaysia, in 2014. The strain was isolated from cloacal swabs and identified based on biochemical characterization (4). Currently, there is neither a complete nor a draft genome sequence of *S*. Typhimurium strain UPM 260 from Malaysia available. The whole-genome sequencing was performed with genomic DNA using paired-end sequencing on the Illumina HiSeq platform. *De novo* assembly was carried out using CLC Genomics Workbench version 7.5.1 (Qiagen, USA), which generated 107 contigs with a G+C content of 52.0% and a total genome size of 4.9 Mb. The sequence was then annotated using the Rapid Annotations using Subsystems Technology (RAST) server (5). The *N*_50_ value was 104,184 bp, and the *L*_50_ was reached in 15 contigs. The data provided will empower further studies of the epidemiology of *S*. Typhimurium strains isolated in Malaysia.

### Accession number(s).

This whole-genome shotgun project has been deposited at NCBI GenBank under the accession number NQWC00000000.
